# Modelling and analysing the coexistence of dual dilemmas in the proactive vaccination game and retroactive treatment game in epidemic viral dynamics

**DOI:** 10.1098/rspa.2019.0484

**Published:** 2019-12-04

**Authors:** K. M. Ariful Kabir, Jun Tanimoto

**Affiliations:** 1 Interdisciplinary Graduate School of Engineering Sciences, Kyushu University, Kasuga-koen, Kasuga-shi, Fukuoka 816-8580, Japan; 2 Faculty of Engineering Sciences, Kyushu University, Kasuga-koen, Kasuga-shi, Fukuoka 816-8580, Japan; 3 Department of Mathematics, Bangladesh University of Engineering and Technology, Dhaka, Bangladesh

**Keywords:** dual dilemma, SITR/V model, antiviral treatment, social efficiency deficit

## Abstract

The dynamics of a spreadable disease are largely governed by four factors: proactive vaccination, retroactive treatment, individual decisions, and the prescribing behaviour of physicians. Under the imposed vaccination policy and antiviral treatment in society, complex factors (costs and expected effects of the vaccines and treatments, and fear of being infected) trigger an emulous situation in which individuals avoid infection by the pre-emptive or *ex post* provision. Aside from the established voluntary vaccination game, we propose a treatment game model associated with the resistance evolution of antiviral/antibiotic overuse. Moreover, the imperfectness of vaccinations has inevitably led to anti-vaccine behaviour, necessitating a proactive treatment policy. However, under the excessively heavy implementation of treatments such as antiviral medicine, resistant strains emerge. The model explicitly exhibits a dual social dilemma situation, in which the treatment behaviour changes on a local time scale, and the vaccination uptake later evolves on a global time scale. The impact of resistance evolution and the coexistence of dual dilemmas are investigated by the control reproduction number and the social efficiency deficit, respectively. Our investigation might elucidate the substantial impacts of both vaccination and treatment in the framework of epidemic dynamics, and hence suggest the appropriate use of antiviral treatment.

## 1. Introduction

The appearance of epidemiological dynamics in the mechanism of pre-emptive voluntary vaccination has been studied in various contexts [1], such as perfect and imperfect vaccination [2,3], dynamical behaviour of vaccination [4], vaccination with information spreading [5], metapopulation migration modelling [6] and heterogeneous networks [7]. Furthermore, Chen & Fu [8] studied an effective antiviral treatment with prescribing behaviour and resistance evolution. Remarkably, influenza-like illnesses, Oseltamivir (Tamiflu) [9] is a widely used *ex post* treatment originally administered against influenza A and B viruses. However, over the years, the societal benefits of antiviral treatment have lessened with overuse, leading to treatment resistance. These trends are evidenced by the interplay between prescription behaviour and resistance evolution. Here, the theoretical studies of vaccination and treatment strategies have considered different effectiveness, associated costs, payoff structures and time scales.

Previously, compartment models with the mean-field approximation, such as the SI [10], SIS [11], SIR [12], SEIR [13] and SEIQR [14] models, are exhibited by dividing the population into several distinct groups. In these designations, S, I, R, E and Q represent the proportions of susceptible, infected, recovered, exposed and quarantined individuals, respectively. Recently, Kabir *et al*. extended the simple SIR model by introducing an awareness effect on epidemic spreading and implemented a two-layer SIR-UA model on well-mixed [15] and heterogeneous networks [16]. Additionally, treatment is an important compartmented state that reduces the disease after infection. Treatments such as antibiotics, quarantine and isolation have been theoretically investigated by many researchers [17–26]. The consequences of vaccination and treatment on an epidemic model were investigated in an influenza model with age structure by Qiu & Feng [27,28] and Feng *et al*. [29], an SIVS model with vaccination age by Li *et al*. [30], an SIR epidemic model with optimal control theory by Zaman *et al*. [31] and a pandemic influenza model by Towers *et al*. [32]. All these works presumed that vaccination, quarantine or treatment would reduce epidemic infection in a simple dynamical situation on local time scales and with no game aspect. By contrast, the present study aims to establish a theoretical epidemic model encompassing both vaccination and treatment as an evolutionary game approach.

The human decision-making process is affected by the cost and risk of the vaccine, self-opinion, networks and neighbours' decisions; therefore, how vaccine acquiescence is influenced by various factors must be investigated [33–48]. According to prior studies, a game approach to epidemiological vaccination can fairly predict the infection risk in both vaccinated and non-vaccinated individuals [49–52]. Such voluntary vaccination game approaches have been studied theoretically and in multi-agent simulations (MAS). To elucidate the mechanism of infectious-disease control, these approaches incorporate a two-layer time scale: a local time scale (epidemic season) of epidemic diffusion and a global time scale on which the strategy updates at the end of the season (at local equilibrium), followed by repeated seasons. Kuga & Tanimoto [53] developed a theoretical model of imperfect vaccination on local and global time scales and validated it by MAS. However, Kabir & Tanimoto [54] claimed that an individual's decision to take a vaccination after social learning (dynamical behaviour) also occurs on local time scales, so this strategy should be updated instantly. Accordingly, it seems that the voluntary vaccination game approach can be implemented into the local time scale while maintaining the framework on the global time scale (strategy update at the end of each season). In the same context, antiviral treatment depends on the local time scale, antiviral resistance and prescribing behaviour.

To shed light on this complex phenomenon, we newly propose the dual-dilemma game structure that considers the roles of both the proactive vaccination and retroactive treatment games. This approach admits different strategy update rules and different time scales of the two provisions. In most of the previous studies [5,6,39–41,49–53], the proactive vaccination permits an individual to accept or decline a vaccination at the end of every epidemic season. This repeated choice is made on global time scales. On the other hand, the retroactive treatment prescribes the behaviour and the antiviral resistance of a certain individual only when s/he is actually infected at a certain time in an epidemic season, which occurs on local time scales. An excessive antiviral treatment may also trigger another viral resistant strain; this behaviour is an expected social problem concerning seasonal influenza in Japan [55]. In this case, the so-called vaccination dilemma modelled by the vaccination game is joined by another dilemma, whereby an individual seeking to use public goods (i.e. accepting the antiviral) induces another viral strain with devastating consequences for others. Using our novel idea backed by the theoretical game approach, we quantify the impact of the pre-emptive vaccination game (before the disease spreading) and the treatment game (after the infection), which includes the prescribing behaviour as an *ex post* provision. Both games are influenced by vaccine effectiveness, treatment efficacy, treatment cost and vaccination cost. Such a double social dilemma situation, perhaps quite ubiquitous in the real world, has not been considered in related previous studies. The vaccination game in the double-dilemma scenario occurs on a larger time scale (over repeated seasons) than the treatment game, which occurs on a day-by-day basis. Therefore, a coevolutionary process can be plausibly modelled in the present study. We employ a pre-emptive control measure that prevents the breakout of infection at an early stage based on an individual's decision. Meanwhile, the *ex post* treatment can be regarded as a fail-safe provision implemented after infection. Owing to the imperfections of vaccines and the unwillingness to take vaccines as a pre-emptive provision, people probably consider a retroactive treatment as the ‘ultimate weapon’ against disease dispersion. However, overuse of antiviral treatment and prescribing behaviour can trigger the emergence of resistant strains, encouraging more *ex post* provision activity by individuals. To handle these two provisions working on different time scales, our model implants the second social dilemma incurred by the antiviral treatment rather than the so-called vaccination dilemma acquired by the proactive provision. To our knowledge, no previous theoretical analysis has considered two provisions in the same context of the evolutionary game process. We also develop another new concept called the *social efficiency deficit* (*SED*). Such a framework can explicitly elucidate the social dual-dilemma on both global (vaccination) and local (treatment) time scales.

In our model, the retroactive antiviral treatment targets the individuals harbouring a sensitive or resistant strain that is controlled by the treatment (antiviral/antibiotic) potency (efficacy). The resistant strain incurs a high medical cost, mortality and the risk of antiviral/antibiotic use, which increases when the demand for antiviral use is driven by the individual's self-interest and overprescribing. Here, we emphasize the social learning behaviour for prescription of antiviral treatment under the evolutionary dynamics of resistance that can uphold the optimal use of the treatment. To explore this evolutionary process of vaccination and treatment, we impose three strategy update rules: individual-based risk assessment (IB-RA), society-based risk assessment (SB-RA) and direct commitment (DC). These rules govern the individual's connection with society. Moreover, we derive the control reproduction numbers of the sensitive and resistant strains, and hence analyse the disease-free equilibria situations in an epidemic.

The remainder of this paper is organized as follows. The ‘Methods and model’ section introduces the new model of epidemic vaccination with the antiviral treatment model and demonstrates it schematically. The ‘Results and discussion’ section validates the proposed model in numerical simulations. Finally, the ‘Conclusion’ section summarizes and further discusses our findings.

## 2. Methods and model

### (a) Basic epidemic dynamics

**Figure 1.**
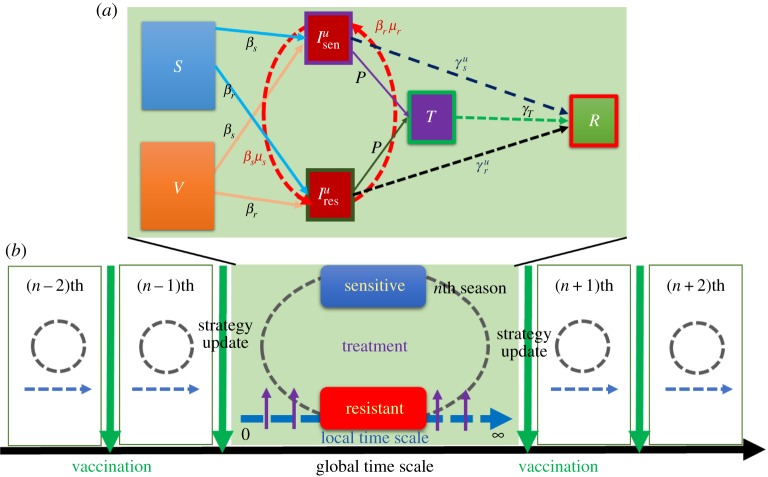
Schematic diagram of (*a*) the SITR/V epidemic model, susceptible and vaccinated individuals get infected at rate *β_s_* for sensitive and *β_r_* for resistant, respectively. The fraction of vaccinated individuals failed to keep immunity by vaccine effectiveness *e*. Sensitive and resistant strains correspondingly performed mutation at rate *μ_s_* and *μ_r_*. The infected individuals may receive treatment with probability *p* and recovered at rate *γ_T_*. However, untreated individuals can be recovered at rates $\gamma_{r}^{u}$ and $\gamma_{s}^{u}$. (*b*) Evolutionary mechanism for both local and global time scale, the evolutionary decision-changing approach takes place on a global time scale (end of each epidemic season) at which individuals can decide whether to vaccinate or not. (Online version in colour.)

To model the social dual-dilemma as a two-stage game, the pre-emptive vaccination and *ex post* treatment are developed in the framework of SIR epidemic dynamics in a well-mixed population (figure 1). In stage 1, the individuals make their vaccination decisions (yes or no) that will control their infection risk during the pandemic season. In stage 2, the infected people with either the sensitive strain or resistant strain decide their treatment provision (treated or untreated) on the local time scale. The antiviral (antibiotic) treatment case incorporates a feedback loop between the prescription behaviour and resistance evolution. To model the disease diffusion in a single season, the initially susceptible people are compartmentalized into vaccinated and non-vaccinated groups. The individuals in the susceptible state can be infected with either sensitive or resistant strains, then seek treatment at an overall treatment rate (treatment probability) denoted as *ωP* , where *P* is the prescribing rate and *ω* determines the probability at which infected people accept treatment from the prescribing individuals. Consequently, the individuals can recover in two ways: natural recovery with no antibiotic/antiviral treatment or recovery after treatment. The epidemiological dynamics are described by the following system of ordinary differential equations

2.1 $$\begin{array}{rl} \dot{S} & =-\beta_{s}S(I_{\text{sen}}^{u}+(1-\varepsilon )T)-\beta_{r}S(I_{\text{res}}^{u}+\varepsilon T), \end{array}$$

2.2 $$\begin{array}{rl} \dot{V} & =-\beta_{s}(V(t)-eV(0))(I_{\text{sen}}^{u}+(1-\varepsilon )T)-\beta_{r}(V(t)-eV(0))(I_{\text{res}}^{u}+\varepsilon T), \end{array}$$

2.3 $$\begin{array}{rl} {\dot{I}}_{\text{sen}}^{u} & =\beta_{s}S(I_{\text{sen}}^{u}+(1-\varepsilon )T)+\beta_{s}(V(t)-eV(0))(I_{\text{sen}}^{u}+(1-\varepsilon )T)-\omega PI_{\text{sen}}^{u}\\ & \;-\beta_{s}\mu_{s}S(I_{\text{sen}}^{u}+(1-\varepsilon )T)+\beta_{r}\mu_{r}S(I_{\text{res}}^{u}+\varepsilon T)-\gamma_{s}I_{\text{sen}}^{u}, \end{array}$$

2.4 $$\begin{array}{rl} {\dot{I}}_{\text{res}}^{u} & =\beta_{r}S(I_{\text{res}}^{u}+\varepsilon T)+\beta_{r}(V(t)-eV(0))(I_{\text{res}}^{u}+\varepsilon T)-\omega PI_{\text{res}}^{u}\\ & \;+\beta_{s}\mu_{s}S(I_{\text{sen}}^{u}+(1-\varepsilon )T)-\beta_{r}\mu_{r}S(I_{\text{res}}^{u}+\varepsilon T)-\gamma_{r}I_{\text{res}}^{u}, \end{array}$$

2.5 $$\begin{array}{rl} \dot{T} & =\omega PI_{\text{sen}}^{u}+\omega PI_{\text{res}}^{u}-\gamma_{T}T \end{array}$$

2.6 $$\begin{array}{rl} \;\text{and}\;\dot{R} & =\gamma_{T}T+\gamma_{s}I_{\text{sen}}^{u}+\gamma_{r}I_{\text{res}}^{u}. \end{array}$$

Here, the fractions of vaccinated plus non-vaccinated individuals infected with the sensitive and resistant strains are denoted by $I_{\text{sen}}^{u}$ and $I_{\text{res}}^{u}$, respectively. *T* denotes the fraction of infected individuals receiving treatment and *R* represents the fraction of individuals who have recovered from infection by a sensitive or resistant strain. In addition, *β_s_* and *β_r_* (*γ_s_* and *γ_r_*) are the disease transmission rates (infection recovery rates) for the sensitive and resistant strains, respectively and *γ_T_* is the recovery rate of infected individuals receiving treatment. Finally, *μ_s_* and *μ_r_* are the mutation rates of the sensitive and resistant strains, respectively. Without mutation, the coexistence of sensitive and resistant strains is forbidden by the competitive exclusion principle. The portion of vaccinated individuals is separated into perfectly immune and non-immune individuals, distinguished by the *vaccine's effectiveness e*(0 ≤ *e* ≤ 1). The treatment efficacy ε controls the treatment efficiency of the sensitive and resistant strains. When *ε* = 0, the treatment is far less beneficial against the resistant strain than against the sensitive strain. On the other hand, when *ε* = 1, the higher number of people in the resistant state is now taking the highest benefit of treatment and is ineffective against the sensitive strain.

### (b) Control reproduction numbers

The basic reproduction number (ratio) $R_{0}$ is the estimated number of infected individuals instigated by a susceptible individual $\left(R_{0}=\beta \text{/}\gamma \right)$. In particular, if $R_{0}<1$, the disease will eventually die out, whereas if $R_{0}>1$, the disease will spread through the population. In this case, we presume separate control reproduction numbers $R_{s}$ and $R_{r}$ for the sensitive and resistant strains, respectively. To evaluate the control reproduction numbers, we initially set *V*(0) ≅ *x*, *S*(0) ≅ 1 − *x* and *μ*_s_, *μ_r_* → 0. From equations (2.3) to (2.5), we obtain

$$\begin{array}{rl} {\dot{I}}_{\text{sen}}^{u} & =\beta_{s}(1-x)(I_{\text{sen}}^{u}+(1-\varepsilon )T)+\beta_{s}(1-e)x(I_{\text{sen}}^{u}+(1-\varepsilon )T)-\omega PI_{\text{sen}}^{u}-0+0-\gamma_{s}I_{\text{sen}}^{u},\\ {\dot{I}}_{\text{res}}^{u} & =\beta_{r}(1-x)(I_{\text{res}}^{u}+\varepsilon T)+\beta_{r}(1-e)x(I_{\text{res}}^{u}+\varepsilon T)-\omega PI_{\text{res}}^{u}-0+0-\gamma_{r}I_{\text{res}}^{u}\\ \;\text{and}\;\dot{T} & =PI_{\text{sen}}^{u}+PI_{\text{res}}^{u}-\gamma_{T}T. \end{array}$$

Let us express the model dynamics as

$$\begin{array}{rl} \left[\begin{array}{c} {\dot{I}}_{\text{sen}}^{u}\\ \dot{T} \end{array}\right] & =\left(\left[\begin{array}{cc} \beta_{s}(1-x)+\beta_{s}(1-e)x & \beta_{s}(1-x)(1-\varepsilon )+\beta_{s}(1-e)x(1-\varepsilon )\\ 0 & 0 \end{array}\right]-\left[\begin{array}{cc} \omega P+\gamma_{s} & 0\\ -\omega P & \gamma_{T} \end{array}\right]\right)\\ & \;\times \left(\begin{array}{c} I_{\text{sen}}^{u}\\ T \end{array}\right). \end{array}$$

Defining

$$F=\left[\begin{array}{cc} \beta_{s}(1-x)+\beta_{s}(1-e)x & \beta_{s}(1-x)(1-\varepsilon )+\beta_{s}(1-e)x(1-\varepsilon )\\ 0 & 0 \end{array}\right],\;V=\left[\begin{array}{cc} \omega P+\gamma_{s} & 0\\ -\omega P & \gamma_{T} \end{array}\right],$$

we can write

2.7 $$FV^{-1}=R_{s}=\frac{\beta_{s}\delta +\beta_{s}\omega P(1-\varepsilon )}{\gamma_{T}(\omega P+\gamma_{s})}((1-x)+(1-e)x).$$

Similarly, we have

2.8 $$R_{r}=\frac{\beta_{r}\delta +\beta_{r}\omega P\varepsilon }{\gamma_{T}(\omega P+\gamma_{r})}((1-x)+(1-e)x).$$

**Figure 2.**
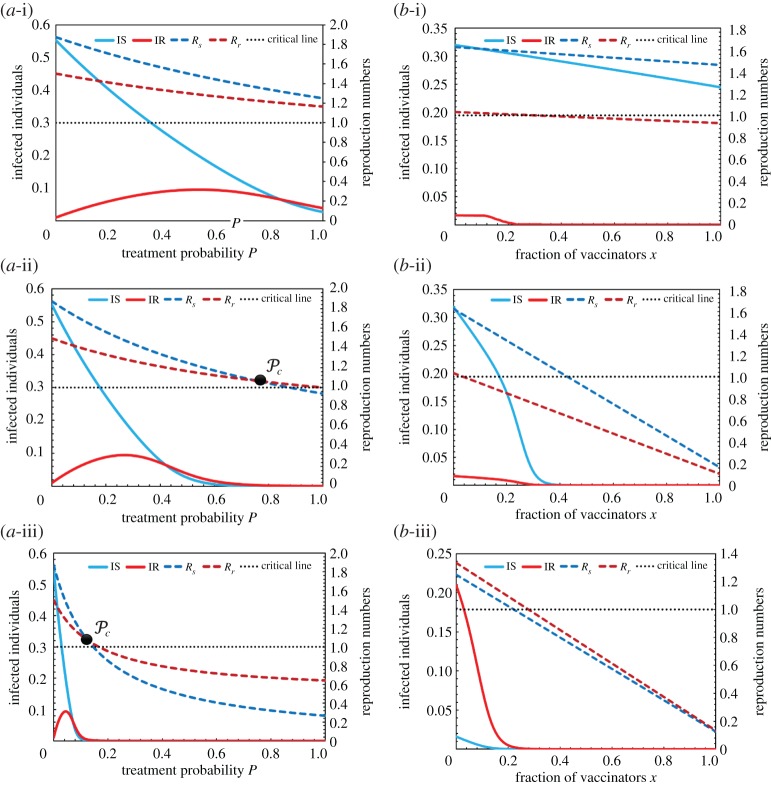
Infection fractions and control reproduction numbers versus (a-*) treatment probability *P* and (b-*) fraction of vaccinators *x*. In panels (a-i)–(a-iii), *ω* = 0.05, *ω* = 0.1 and *ω* = 0.6, respectively. In panels (b-i)–(b-iii), the parameter set (*e*, *ε*) = (0.1, 0.1), (0.9, 0.1) and (0.9, 1.0), respectively. The other parameter values are *β_s_* = 0.25, *β_r_* = 0.2, *γ_s_* = *γ_r_* = 0.1, *μ_s_* = *μ_r_* = 10^−4^, *γ_T_* = 0.3, (in a-*) *e* = 0.5, *X* = 0.5 and *P* = 0.5, *ω* = 0.15 (in b-*). (Online version in colour.)

As mentioned above, the control reproduction number must reflect the stability of the disease-free equilibrium state. According to equations (2.7) and (2.8), the control reproduction numbers of both sensitive $\left(R_{s}\right)$ and resistant $\left(R_{r}\right)$ strains act like a decreasing function of *P* and *x*; both ranging from 0 to 1 displayed in figure 2. The left panels of figure 2 (panel a-*) plot the line graphs of infected individuals versus the treatment probability *P* for the sensitive (IS) and resistant (IR) strains for different values of *ω* = 0.05, 0.1 and 0.6.

We now examine the simulation results of equations (2.1)–(2.8), and hence find the critical *P* of the maximal potential antiviral treatment beyond which $R_{r}$ is not going beyond $R_{s}$. Here, the proportion of vaccinated individuals is assumed constant (*x* = 0.5) [5,6,53]. The experimental results in the left panels of figure 2 are summarized below:

(a) Panel (a-i): When the probability of treatment acceptance *ω* was low (0.05), the antiviral treatment did not effectively eradicate the disease and the sensitive strain always dominated the resistant strain.
(b) Panel (a-ii): When *ω* increased to 0.1, we observed a critical intersection point ($P_{c}$) at which the control reproduction number of the sensitive strain equalled that of the resistant strain.
(c) Panel (a-iii): When the probability of treatment acceptance exceeded the probability of treatment refusal (*ω* = 0.6), the control reproduction number of both strains could be less than 1, indicating that treatment could fully eradicate the disease. At relatively high *ω*, the individuals will likely be treated when infected.

In panels (a-ii) and (a-iii), we can find the critical treatment probability $P_{c}$ at which the treatment probabilities of the sensitive and resistant strains are equal. When $0<P<P_{c}$, the sensitive strain is more prevalent than the resistant strain (i.e. $R_{s}>R_{r}$). However, when $P>P_{c}\;$, the resistant strain will outperform against the sensitive strain. At the social optimum, the critical treatment probability $P_{c}$ specifies the maximum treatment control under which resistant strains will not emerge. Assuming $R_{s}=R_{r}$ in equations (2.7) and (2.8), $P_{c}$ is calculated as

2.9 $$P_{c}=\frac{\beta_{s}\delta -\beta_{r}\delta }{\beta_{r}\omega \varepsilon -\beta_{s}\omega (1-\varepsilon )}.$$

Right panels (b-i), (b-ii) and (b-iii) of figure 2 plot the vaccine effectiveness and treatment efficacy versus fraction of vaccinators *x* for different combinations of the control reproduction numbers ($R_{s}$ and $R_{r}$) and infected individuals (IS and IR): (*e* = 0.1, *ε* = 0.1), (*e* = 0.9, *ε* = 0.1) and (*e* = 0.9, *ε* = 1.0), respectively. Comparing panels (b-i) and (b-ii), increasing *e* enhances the inclination of $R_{s}$ and $R_{r}$, indicating a highly reliable vaccine that rapidly eradicates the disease. Also, low treatment efficacy (*ε* = 0.1) encourages the sensitive strain, whereas the resistant strain presents in the almost disease-free situation. On the other hand, at the highest treatment efficacy (*ε* = 1.0; panel (b-iii)), the sensitive strain dominates the resistant strain. Both strains become disease-free as the vaccine effectiveness (i.e. *x*) increases.

### (c) Coevolutionary dynamics

We incorporate two-game aspects (treatment and vaccination) in a single epidemiological game model. This model reproduces the coevolutions of accepting a vaccination at the beginning of every season and receiving treatment after becoming infected in a season. In the treatment game, the individual decision to receive or decline treatment against the infectious sensitive and resistant strains occurs on the local time scale. In the vaccination game, the individuals can consent to alter their strategy (accept or decline vaccination) based on the progress of the last pandemic season, which occurs on the global time scale.

#### (i) Treatment game

Based on a feedback loop between the resistance evolution and prescription norm, the game approach establishes a social learning dynamical process that somehow controls the optimum use of the antiviral treatment. To quantify the evolutionary decision dynamics of treatment versus non-treatment (prescribing versus non-prescribing), we specify the relative treatment cost *C_T_* vis-à-vis the infection cost *C_i_* = 1. We also introduce the benefit *B_T_* of treating the sensitive strain (the resistant strain is excluded, because it is much more difficult to treat than the sensitive strain, so *B_T_* is always positive). This idea is formulated as a two-strategy game in table 1.

**Table 1.** Payoff structure of the treatment game with four costs.

strategy	sensitive	resistant
treated (prescribing)	*B_T_* − *C_T_* − 1	−*C_T_* − 1
untreated (non-prescribing)	*B_T_* − 1	−1

The fractions of individuals infected with the sensitive and resistant strains are, respectively, given by

2.10.1 $$f_{s}=\beta_{s}S(I_{\text{sen}}^{u}+(1-\varepsilon )T)+\beta_{s}(V(t)-eV(0))(I_{\text{sen}}^{u}+(1-\varepsilon )T$$

and

2.10.2 $$f_{r}=\beta_{r}S(I_{\text{res}}^{u}+\varepsilon T)+\beta_{r}(V(t)-eV(0))(I_{\text{res}}^{u}+\varepsilon T).$$

The expected payoffs of the treated and untreated individuals are, respectively, given by

2.11.1 $$\langle \pi_{T}\rangle =\frac{f_{s}(B_{T}-C_{T}-1)+f_{r}(-C_{T}-1)}{f_{s}+f_{r}}$$

and

2.11.2 $$\langle \pi_{U}\rangle =\frac{f_{s}(B_{T}-1)+f_{r}(-1)}{f_{s}+f_{r}}.$$

To model the two-strategy game, we presume the DC strategy update rule presented in Iwamura & Tanimoto [51]. This rule is designed by comparing the expected payoffs of the treated $\left\langle \pi_{T}\right\rangle$ and untreated $\left\langle \pi_{U}\right\rangle$ individuals. In the present study, the strategy updates (in both the treatment and vaccination games (see later)) apply the mean-field approximation. The modified Fermi function of DC is given by

2.12 $$\text{Prob}(s_{i}\leftarrow s_{j})=\frac{1}{1+\text{exp}\left[-(\left\langle \pi_{j}\rangle -\langle \pi_{i}\right\rangle )/\kappa \right]},$$

where $\left\langle \pi_{i}\right\rangle$ and $\left\langle \pi_{j}\right\rangle$ are the mean payoffs of the focal portion of individuals and the opponent strategy (fraction), respectively. Here, we consider pairwise comparison between two groups; which depends on the payoff difference $\left\langle \pi_{j}\rangle -\langle \pi_{i}\right\rangle$. Because, the pairwise fermi has been well-accepted strategy-updating procedure that stochastically comparable to the real-world human decision-making process. The probabilities of the population transiting from untreated to treated and from treated to untreated are, respectively, calculated as

2.13.1 $$\text{Prob}(T\leftarrow U)=\frac{1}{1+\text{exp}\left[-(\left\langle \pi_{U}\rangle -\langle \pi_{T}\right\rangle )\text{/}\kappa \right]}$$

and

2.13.2 $$\text{Prob}(U\leftarrow T)=\frac{1}{1+\text{exp}\left[-\left(\left\langle \pi_{T}\rangle -\langle \pi_{U}\right\rangle \right)/\kappa \right]}.$$

Consequently, the treatment game is expressed by the following DC dynamics:

2.14 $$\dot{P}=-P(t)\cdot \text{Pro}b(T\leftarrow U)+(1-P(t))\cdot \text{Prob}(U\leftarrow T).$$

#### (ii) Vaccination game

This subsection models the evolutionary strategy update rule, considering the individual decision-making on vaccination versus non-vaccination in the global time scale. The vaccination cost *C_r_* is defined relative to the disease cost *C_i_* = 1. As shown in table 2, we presume four portions of individuals in the equilibrium state (end of each season): (i) healthy and vaccinated (HV); individuals paying −*C*_*r*_, (ii) infected and vaccinated (IV); individuals paying −*C_r_* − 1, (iii) healthy and non-vaccinated (successful free rider (SFR)); individuals paying nothing, and (iv) infected and non-vaccinated (successful free rider (FFR)); individuals paying − 1. The fractions of all (whether healthy or unhealthy) vaccinated and non-vaccinated individuals are denoted as *V* and NV***,*** respectively.

**Table 2.** Individual payoff structures and fractions of individuals at equilibrium.

	healthy and	infected and	healthy and	infected and
strategy	vaccinated	vaccinated	non-vaccinated	non-vaccinated
payoff	−*C_r_*	−*C_r_* − 1	0	−1
fraction of individuals	HV	IV	SFR	FFR
	V = HV + IV		NV = SFR + FFR	

Considering the defined payoff structure and the portion of individuals presented in table 2, the social average payoff 〈π〉, expected value of vaccinators 〈*π_C_*〉 and expected value of non-vaccinators 〈*π_D_*〉 are, respectively, given by

2.15 $$\begin{array}{rl} \left\langle \pi \right\rangle & =-C_{r}\cdot \text{HV}(\tau )-(C_{r}+1)\cdot \text{IV}(\tau )-\text{INV}(\tau ), \end{array}$$

2.16 $$\begin{array}{rl} \left\langle \pi_{C}\right\rangle & =\frac{\left\{-C_{r}\cdot \text{HV}(\tau )-(C_{r}+1)\cdot \text{IV}(\tau )\right\}}{\left\{\text{HV}(\tau )+\text{IV}(\tau )\right\}} \end{array}$$

2.17 $$\begin{array}{rl} \;\text{and}\;\langle \pi_{D}\;\rangle & =\frac{\left\{-\text{INV}(\tau )\right\}}{\left\{\text{HNV}(\tau )+\text{INV}(\tau )\right\}}. \end{array}$$

To formulate the evolutionary process, we consider two types of strategy adaptation procedures [53]; IB-RA and SB-RA. In the case of IB-RA, an individual can update strategy by observing a neighbour's strategy. The update is governed by the transition probability Prob(*s_i_* ← *s_j_*) taken from the pairwise Fermi function [1]. Alternatively, in an SB-RA, an individual relies on the mean payoff of all opposite neighbours [2]. We apply the mean-field approximation to formulate the adaptation dynamics in both the IB-RA and SB-RA rules. Here, we replace the first row by the actual transition probabilities in the second row of table 3.

**Table 3.** Transition probabilities of all possible cases for IB-RA and SB-RA.

	IB-RA	SB-RA
PW-Fermi	Prob(si←sj)=1∫1+exp[−(πj−πi)/κ]	Prob(si←sj)=1∫1+exp[−(⟨πj⟩−πi)/κ]∫
transition probability	Prob(HV←SFR)=1∫1+exp[−(0−(−Cr))/κ]	Prob(HV←NV)=1∫1+exp[−(⟨πD⟩−(−Cr))/κ]∫
	Prob(HV←FFR)=1∫1+exp[−(−1−(−Cr))/κ],	Prob(IV←NV)=1∫1+exp[−(⟨πD⟩−(−Cr−1))/κ]∫
	Prob(IV←SFR)=1∫1+exp[−(0−(−Cr−1))/κ],	Prob(SFR←V)=1∫1+exp[−(⟨πC⟩−0)/κ]∫
	Prob(IV←FFR)=1∫1+exp[−(−1−(−Cr−1))/κ]	Prob(FFR←V)=1∫1+exp[−(⟨πC⟩−(−1))/κ]∫
	Prob(SFR←HV)=1∫1+exp[−(−Cr−0)/κ]∫,	
	Prob(FFR←HV)=1∫1+exp[−(−Cr−(−1))/κ]∫,	
	Prob(SFR←IV)=1∫1+exp[−(−Cr−1−0)/κ]∫,	
	Prob(FFR←IV)=1∫1+exp[−(−Cr−1−(−1))/κ]∫	

#### (iii) Evolutionary dynamical system for the vaccination game

To establish the dynamical system at the end of each epidemic season, we formulate IB-RA and SB-RA as mathematical models that modify the fraction of vaccinators *x*. The evolutionary dynamics of the IB-RA and SB-RA are, respectively, given by

Individual-based risk assessment (IB-RA)

2.18 $$\begin{array}{rl} \dot{x} & =\text{HV}(x,\infty )\cdot \text{SFR}(x,\infty )\cdot (\text{Prob}(\text{SFR}\leftarrow \text{HV})-\text{Prob}(\text{HV}\leftarrow \text{SFR}))\\ & \;+\text{HV}(x,\infty )\cdot \text{FFR}(x,\infty )\cdot (\text{Prob}(\text{FFR}\leftarrow \mathrm{HV})-\text{Prob}(\text{HV}\leftarrow \text{FFR}))\\ & \;+\text{IV}(x,\infty )\cdot \text{HNV}(x,\infty )\cdot (\text{Prob}(\text{SFR}\leftarrow \mathrm{IV})-\text{Prob}(\text{IV}\leftarrow \text{SFR}))\\ & \;+\text{IV}(x,\infty )\cdot \text{FFR}(x,\infty )\cdot (\text{Prob}(\text{FFR}\leftarrow \text{IV})-\mathrm{Prob}(\text{IV}\leftarrow \text{FFR}))\}. \end{array}$$

Society-based risk assessment (SB-RA)

2.19 $$\begin{array}{rl} \dot{x} & =-(1-x)\cdot \text{HV}(x,\infty )\cdot \text{Prob}(\text{HV}\leftarrow \text{NV})-(1-x)\cdot \text{IV}(x,\infty )\cdot \text{Prob}(\text{IV}\leftarrow \text{NV})\\ & \;+x\cdot \text{SFR}(x,\infty )\cdot \text{Prob}(\text{SFR}\leftarrow V)-x\cdot \text{FFR}(x,\infty )\cdot \text{Prob}(\text{FFR}\leftarrow V). \end{array}$$

We have now established all mathematical frameworks in both the vaccination and treatment cases. The above set of equations is numerically solved by the explicit finite difference method. The calculation results affected by the two-stage process (the SITR/V dynamical model and treatment update) are together obtained in a single season (local update) at equilibrium, and the vaccination strategy is adopted at the end of every season (global update). The initial values were set as *V*(*x*, 0) = *x*, *S*(*x*, 0) ≈ 1 − *x*, $I_{\text{sen}}^{u}(x,0)\approx 0$, $I_{\mathrm{res}}^{u}(x,0)\approx 0$, *T*(*x*, 0) = 0 and *R*(*x*, 0) = 0.

## 3. Results and discussion

The results are presented in two-dimensional (2D) phase diagrams. The strategy update rules of the vaccination game (IB-RA or SB-RA) and the DC were applied on the global and local time scales, respectively (where the DC depicts the prescribing behaviour of the treatment policy). The coalescing impact of proactive vaccination and the retroactive treatment policy based on human behaviour was formulated by the conventional mean-field approximation. The simultaneous changes of two coevolutionary decision-making processes were globally demonstrated in two cases. We first explored the phase diagram of the final epidemic size (FES), vaccination coverage (VC), fraction of treated people (FTR) and the average social payoff (ASP) while varying two parameters: the vaccination effectiveness *e* and the treatment efficacy *ε*, maintaining sensible fixed values of the other parameters. In the second case, we introduce SED that explicitly reveals the underlying social dilemmas in the vaccination and treatment games. In the numerical simulation, we set the social-benefit cost *B_T_* = 0.3, the infection rates as *β_s_* = 0.25and *β_r_* = 0.20, the recovery rates as *γ_s_* = 0.1, *γ_r_* = 0.1 and *γ_T_* = 0.3, and the mutation rates as *μ_s_* = 0.00001 and *μ_r_* = 0.00001.

**Figure 3.**
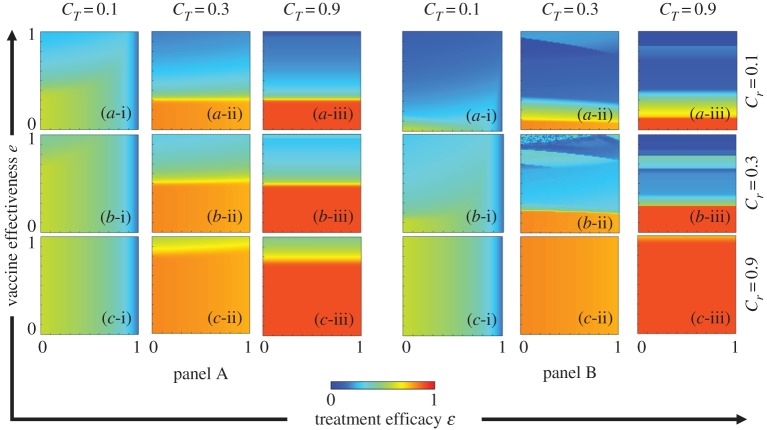
Phase diagrams of FES. Panels A and B plot the results of IB-RA and SB-RA, respectively. In both panels, the first, second and third rows display the results of varying the vaccination costs: (a-*) *C_r_* = 0.1, (b-*) *C_r_* = 0.3 and (c-*) *C_r_* = 0.9. Meanwhile, the first, second and third columns show the results of varying the treatment costs (*-i) *C_T_* = 0.1, (*-ii) *C_T_* = 0.3 and (*-iii) *C_T_* = 0.9. The other parameters are *β_s_* = 0.25, *β_r_* = 0.2, *γ_s_* = *γ_r_* = 0.1, *μ_s_* = *μ_r_* = 10^−4^, *γ_T_* = 0.3 and *ω* = 0.15. (Online version in colour.)

**Figure 4.**
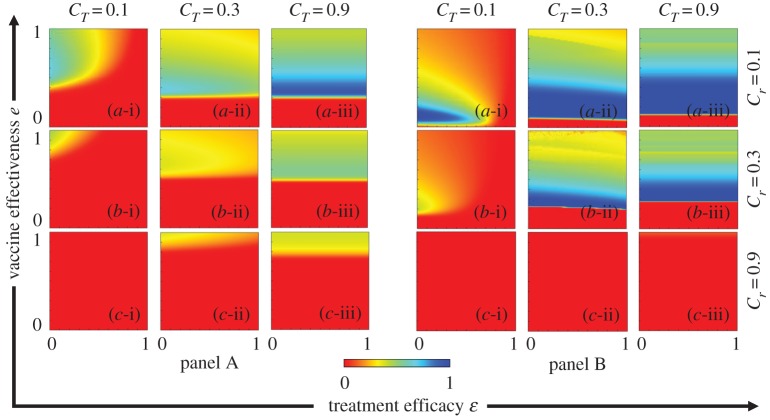
Phase diagrams of VC. Panels A and B plot the results of IB-RA and SB-RA, respectively. In both panels, the first, second and third rows display the results of varying the vaccination costs: (a-*) *C_r_* = 0.1, (b-*) *C_r_* = 0.3 and (c-*) *C_r_* = 0.9. Meanwhile, the first, second and third columns show the results of varying the treatment costs: (*-i) *C_T_* = 0.1, (*-ii) *C_T_* = 0.3 and (*-iii) *C_T_* = 0.9. The other parameters are *β_s_* = 0.25, *β_r_* = 0.2, *γ_s_* = *γ_r_* = 0.1, *μ_s_* = *μ_r_* = 10^−4^, *γ_T_* = 0.3 and *ω* = 0.15. (Online version in colour.)

**Figure 5.**
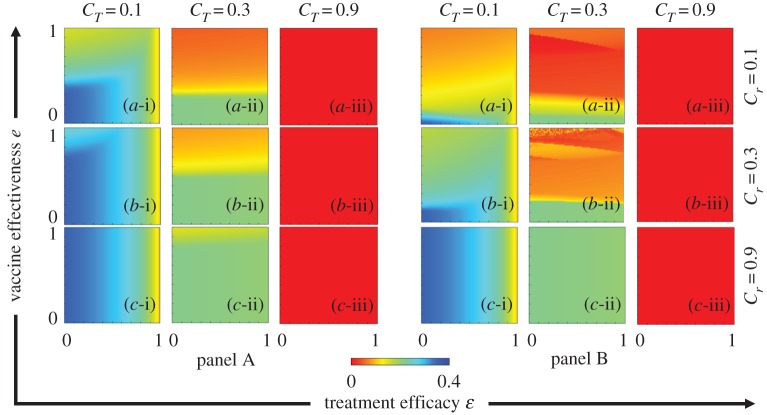
Phase diagrams of FTR. Panels A and B plot the results of IB-RA and SB-RA, respectively. In both panels, the first, second and third rows display the results of varying the vaccination costs: (a-*) *C_r_* = 0.1, (b-*) *C_r_* = 0.3 and (c-*) *C_r_* = 0.9. Meanwhile, the first, second and third columns show the results of varying the treatment costs: (*-i) *C_T_* = 0.1, (*-ii) *C_T_* = 0.3 and (*-iii) *C_T_* = 0.9. The other parameters are *β_s_* = 0.25, *β_r_* = 0.2, *γ_s_* = *γ_r_* = 0.1, *μ_s_* = *μ_r_* = 10^−4^, *γ_T_* = 0.3 and *ω* = 0.15. (Online version in colour.)

**Figure 6.**
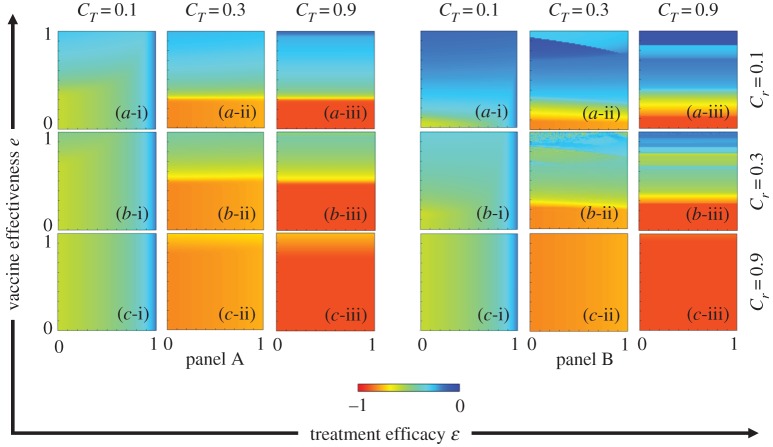
Phase diagram of ASP. Panels A and B plot the results of IB-RA and SB-RA, respectively. In both panels, the first, second and third rows display the results of varying the vaccination costs: (a-*) *C_r_* = 0.1, (b-*) *C_r_* = 0.3 and (c-*) *C_r_* = 0.9. Meanwhile, the first, second and third columns show the results of varying the treatment costs: (*-i) *C_T_* = 0.1, (*-ii) *C_T_* = 0.3 and (*-iii) *C_T_* = 0.9. The other parameters are *β_s_* = 0.25, *β_r_* = 0.2, *γ_s_* = *γ_r_* = 0.1, *μ_s_* = *μ_r_* = 10^−4^, *γ_T_* = 0.3 and *ω* = 0.15. (Online version in colour.)

To elucidate the mechanism of the epidemic spreading process and the interplay between vaccination and treatment, we plotted the FES, VC, FTR and ASP in the *e* versus *ε*. The results are plotted in figures 3–6, respectively. In each figure, panel sets A and B display the IB-RA and SB-RA case, respectively. Moreover, panels (*-i), (*-ii) and (*-iii) show the results under treatment costs *C_T_* = 0.1, 0.3 and 0.9, respectively, whereas panels (a-*), (b-*) and (c-*) present the results under vaccination costs *C_r_* = 0.1, 0.3 and 0.9, respectively.

As shown in figure 3, the FES increased (higher infection region) with increasing vaccination cost *C_r_* and treatment cost *C_T_*. Reducing the cost of both vaccination (*C_r_* = 0.1) and treatment (*C_T_* = 0.1) improved the FES (lowered the infection region) (figure 3(a-i), both panels). Furthermore, reducing the costs (*C_r_*, *C_T_* < 0.5) more effectively benefitted the FES (in terms of the critical borderline between infection breakout and diminution) in IB-RA than in SB-RA (cf. panel sets A and B); the reverse tendency was found at higher costs (*C_r_*, *C_T_*) as analogously reported by Fukuda *et al*. [2]. When the costs are relatively low, vaccination is more encouraged in SB-RA than in IB-RA, which hampers the reduction in the infected number of individuals in the early stage of each season. Thus, based on the human decision-making of whether to accept or decline both vaccination and treatment, the changing propensity of the FES can be significantly enhanced by the vaccination effectiveness, treatment efficacy and their corresponding costs.

As indicated in figure 4, lowering the vaccination cost and increasing the reliability (effectiveness) of the vaccine enticed the individuals to accept more vaccines. This tendency was more marked in the SB-RA than in the IB-RA, and enhanced the vaccination acceptance when the post-infection treatment cost was high (*C_T_* = 0.9) on the local time scale. However, the portion of individuals making treatment provision (FTR) diminished at higher treatment costs (*C_T_* = 0.9) in both schemes (panels (*-iii) in figure 5). A small treatment cost attracts individuals to the treatment provision, whereas a higher cost hampers the treatment-seeking behaviour (lowers the FTR). Therefore, either lowering the vaccination cost or improving the vaccination effectiveness will improve the FTR. Meanwhile, reducing the treatment cost increased the FTR even when the vaccination cost was high (Panels (*-i) of figure 5). Briefly, both the vaccination and treatment costs significantly influence the individual choice. The vaccine effectiveness and treatment efficacy also play substantial roles. The above results are consolidated by the ASP results plotted in figure 6.

### (a) Dual-dilemma structure

**Figure 7.**
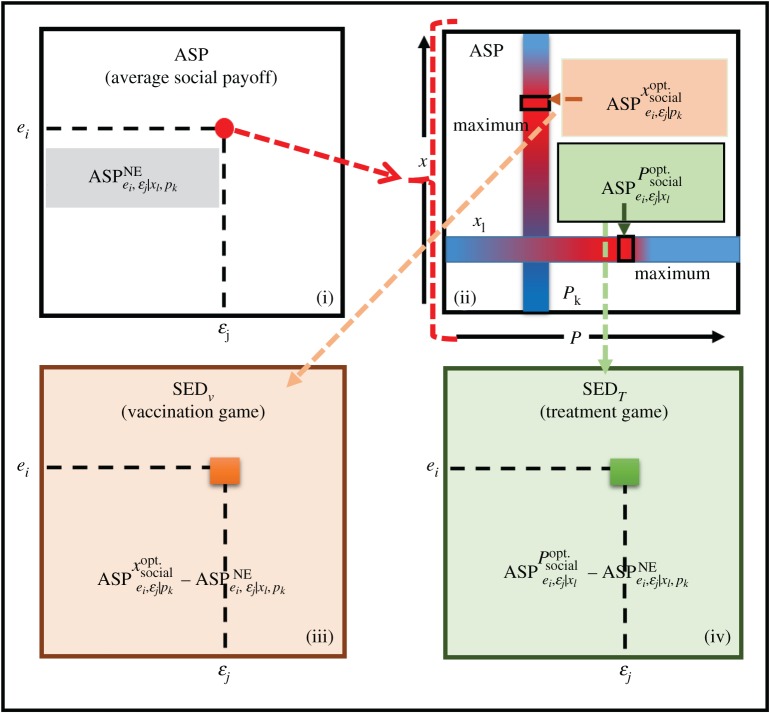
Flow diagram of the SED. (i) In the ASP phase diagram in the vaccination and treatment games, the NE point is evaluated at every set of points (*e*, *ε*). (ii) The optimum *x* and *P-*values are calculated in the default ASP scenario (no game). (iii) SED in the vaccination game, denoted by SED*_v_*, and (iv) SED in the treatment game, denoted by SED*_T_*. (Online version in colour.)

To explore the dual-dilemma structure on an epidemiological model, we considered the joint impact of vaccination and treatment games in the same context. A typical scenario is demonstrated in figure 7. Our idea was motivated by the endorsement of social dilemma situations in the strategies of evolutionary game theory, in which the players are all individuals in a well-mixed population. In a coevolutionary adaptation process, it is important to know whether the social dual-dilemma exists under certain combinations of the model parameters, such as the vaccination effectiveness, treatment efficiency and their associated costs. Unlike simple 2 by 2 games in which the so-called dilemma strength (DS) can be explicitly defined [1], a real social dilemma typically observed in the vaccination game [2,5–7,36–44], traffic flow [46–52] and others may have a time-variable game structure. In the vaccination and traffic games, this structure is mainly influenced by the disease-spreading and traffic flow dynamics, respectively. The time-variable game structure is too complex to represent by the payoff matrix in a 2 × 2 game or the time-constant payoff structure function in the 2-strategy and *n*-player game. Thus, the DS is difficult to determine in advance even when the mathematical model is well defined. Our new SED concept (defined above and mathematically formulated blow) was inspired by the seminal idea of traffic flow analysis (e.g. [56–62]). The DS indicates the existence or absence of a social dilemma (behaving as a prognostic index), whereas the SED provides an ‘*ex post*’ or diagnostic index. Here, let us define SED as the gap between the result of the evolutionary trail (which can be evaluated by the Nash equilibrium (NE)) and the optimum solution (ideal result from a social-welfare standpoint [63]). The payoff at the NE can always be observed by taking an evolutionary game approach, whereas the optimal social payoff is observable in a model of any complexity. Therefore, one can evaluate the SED in any context, and hence predict the occurrence of a social dilemma (if the SED is positive, the gap exists; if it is zero, the evolutionary trail matches the optimum). Thus, the SED indicates that the payoff can be improved from that at the NE. Mathematically, the SED is given by

3.1 SED=(social optimal payoff) − (payoff at Nash equilibrium)

Again, let us reiterate that SED = 0 implies no social dilemma, while any social dilemma causes a positive SED. According to the abovementioned conceptual definition, SED in the current model (which deals with both vaccination and treatment games) is given by

3.2.1 $$\text{SED}=\frac{{\text{ASP}}_{\text{social}}^{\text{Opt}}-\text{AS}{\text{P}}^{\text{NE}}}{C_{i}}={\text{ASP}}_{\text{social}}^{\text{Opt}}-\text{AS}{\text{P}}^{\text{NE}}.$$

Meanwhile, the ASP is the quantity of payoff. The superscript ‘Opt’ and subscript ‘social’ together indicate the social optimal. The *C_i_* was taken as the standardizing denominator as previously defined as 1. The SEDs in the vaccination and treatment game of the present model are, respectively, defined as follows:

3.2.2 $$\text{SE}{\text{D}}_{V}={\text{ASP}}_{e_{i},\varepsilon_{j}|p_{k}}^{x_{\text{social}}^{\text{opt}\text{.}}}-{\text{ASP}}_{e_{i},\varepsilon_{j}|x_{l},p_{k}}^{\text{NE}}$$

and

3.2.3 $$\text{SE}{\text{D}}_{T}={\text{ASP}}_{e_{i},\varepsilon_{j}|x_{l}}^{P_{\text{social}}^{\text{opt}\text{.}}}-{\text{ASP}}_{e_{i},\varepsilon_{j}|x_{l},p_{k}}^{\text{NE}}$$

where ${\text{ASP}}_{e_{i},\varepsilon_{j}|x_{l},p_{k}}^{\text{NE}}$ indicates the average social payoff at the NE, estimated when both games (vaccination and treatment) have occurred together on the local and global time scales. Now, to understand the ${\text{ASP}}_{e_{i},\varepsilon_{j}|p_{k}}^{{\text{x}}_{\text{social}}^{\text{opt}\text{.}}}$ and ${\text{ASP}}_{e_{i},\varepsilon_{j}|x_{l}}^{P_{\text{social}}^{\text{opt}\text{.}}}$ the terms $x_{\text{social}}^{\text{opt}\text{.}}$ and $P_{\text{social}}^{\text{opt}\text{.}}$ reflect the fact that the maximum ASP is obtained for varying *x* ranging from 0 to 1\ (for fixed *p_k_*) and varying *P* from 0 to 1(for fixed *x_l_*), respectively. Figure 7 presents the stepwise procedure of finding SED and quantifying the dual dilemma in the proposed method.

*Step 1.* Construct the ASP phase diagram based on the evolutionary game approach (figure 7(i)). To this end, implement both the vaccination and treatment games and obtain the appropriate ASP at the NE, along with a certain vaccine effectiveness (*e*) and treatment efficacy (*ε*). The ASP associated with the fraction of vaccinators and probability of treatment (*x_l_*, *p_k_*) at the NE can also be observed for a precise (*e_i_*, *ε_j_*).
*Step 2.* For this fixed (*e_i_*, *ε_j_*), evaluate ${\text{ASP}}_{e_{i},\varepsilon_{j}|p_{k}}^{x_{\text{social}}^{\text{opt}\text{.}}}$ while constraining *P* at *p_k_* and the maximal ASP is evaluated by varying *x* under the default setting. Likewise evaluate ${\text{ASP}}_{e_{i},\varepsilon_{j}|x_{l}}^{P_{\text{social}}^{\text{opt}}}$ while constraining *x* at *x*_*l*_ (figure 7(ii)).
*Step 3.* Using the values obtained in Steps 1 and 2, calculate the values of SED_V_ and SED_T_ formulated in equations (3.2.2) and (3.2.3) (figure 7(iii),(iv), respectively). The existence of a social dilemma in the model can be visually depicted in a heat-map of the SEDs.

**Figure 8.**
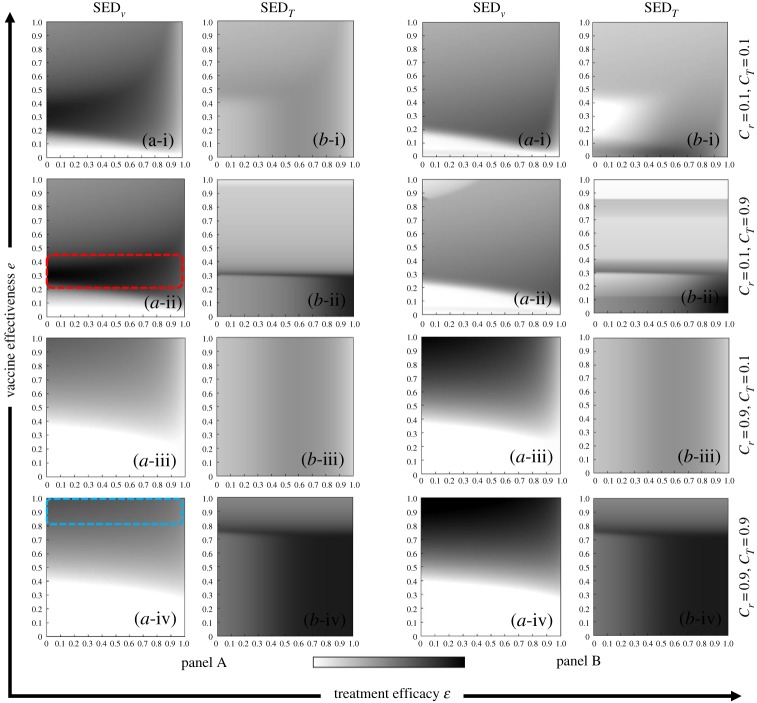
Phase diagram of SED, panels A and B present the results of IB-RA and SB-RA, respectively. In both panels, rows 1–4 display the vaccination and treatment costs (*C_r_*, *C_T_*) as (*-i) (0.1, 0.1), (*-ii) (0.1, 0.9), (*-iii) (0.9, 0.1) and (*-iv) (0.9, 0.9). Meanwhile, the first and second columns of both panels show (a-*) SED*_v_* and (b-*) SED*_T_*.

To address whether a dual dilemma exists in the vaccination and treatment games, we plot the SED diagrams in the vaccination effectiveness (*e*) versus treatment efficacy (*ε*) planes at different costs. Panels A and B of figure 8 are plotted under the strategy update rules IB-RA and SB-RA, respectively, and plots (a-*) and (b-*) present the SEDs in the vaccination game SED_v_ and the treatment game SED_T_, respectively. Moreover, the combined vaccination and treatment costs (*C_r_*, *C_T_*) were varied as (0.1, 0.1), (0.1, 0.9), (0.9, 0.1) (0.1, 0.1), and (0.9, 0.9) in plots (*-i), (*-ii), (*-iii) and (*-iv), respectively. As demonstrated in the above SED formulation, the dilemma situation (non-white areas in the plots) appeared in all cases, but interestingly depended on *e* and *ε*. No-dilemma regions, in which either the vaccination or treatment game became trivial, were also observed. Now, comparing panels (a-i) and (a-ii) for *C_r_* = 0.1 with panels (a-iii) and (a-iv) for *C_r_* = 0.9, one finds that the non-dilemma region (whiteout region) expanded with increasing vaccination cost. At the smaller vaccination cost (*C_r_* = 0.1), the region of larger SED appeared at a relatively low vaccination effectiveness (around *e* = 0.4; dotted red box in panel (a-i)). The huge gap between NE and the social optimal results from the lower NE due to a lower efficiency which makes fewer people commit vaccination when compared with a situation allowing a relatively higher efficiency which makes much more people commit. By contrast, a relatively high vaccination efficiency entices people to vaccinate, thus increasing the NE. Meanwhile, increasing the vaccination cost (*C_r_* = 0.9) shifted the region of reasonably high SED to the maximally high vaccination effectiveness (dotted blue box in panel (a-iv)). This occurred because despite the high vaccination effectiveness, low vaccination efficiency hampers the commitment to vaccination. Reliable vaccination provides a high commitment incentive, but high cost encourages free-riding on the herd immunity of the devoted others. In summary, we have numerically demonstrated that the SED precisely and easily detects the social dilemma in our dual-dilemma coevolutionary model.

## 4. Conclusion

This paper developed an SITR/V epidemic model that combines the effects of proactive vaccination and retroactive treatment on the control and prevention of infectious viral diseases. The model building and its investigation were presented in this work. The most important contribution is that our new model gives a brand-new framework in which both pre-emptive vaccination and treatment as an *ex post* provision having different evolutionary time scales, which dovetails the ideological dynamics with the dynamics of the human decision-making process. This concept has been never studied. Also, our model gives a clear procedure to quantify the social dilemmas, respectively, entailed by ‘vaccination game’ and ‘treatment game’.

The most novel aspect of our model is the simultaneous implementation of two social dilemma games: the antiviral treatment game and the vaccination game, which none of the previous work has tried to implement. The vaccination game implements on the global time scale under two strategy adaptation rules: IB-RA and SB-RA, assuming an infinite and well-mixed population. Meanwhile, the treatment game describes the behaviour of antiviral administration with resistant-strain emergence. The treatment game is updated on the local time scale by presuming the DC rule, and precisely integrating a feedback loop between the sensitive and resistant strains. The outcome of antiviral and vaccination use depends on the effectiveness of the vaccine, the efficiency of treatment and their corresponding costs. Increasing the effectiveness of the vaccine and lowering its administration cost reduced the final epidemic size (increased vaccination coverage). Lowering the treatment cost and enhancing the treatment efficacy exerted a similar effect. Thus, by applying retroactive antiviral use with pre-emptive vaccination, we can deeply understand and investigate individual decisions regarding vaccination and implement proper strategies that lessen the diffusion of infection or recommend appropriate and careful administration of both antivirals and vaccination. We also introduced the social optimum point $P_{c}$ that distinguishes the conditions under which treatment resistance emerges under antibiotic overuse and its associated factors.

Besides evolving the voluntary vaccination game, our model introduces a new game aspect with two provisions: vaccination as a proactive measure and treatment as a retroactive measure. Presuming seasonal influenza-like diseases, the (pre-emptive) vaccination works over repeated seasons on global time scales, whereas the *ex post* treatment works seasonally on local time scales and depends on the antiviral cost, prescription behaviour and resistant-strain emergence. In the present model, we successfully established a ‘double-layer’ game structure of pre-emptive vaccination and *ex post* treatment. Unlike the vaccination game model, which only considers whether the vaccine is accepted or declined, and whether an *ex post* provision is taken in a single season, the treatment game includes an aspect that depends on the antiviral resistance evolution and prescribing behaviour. To explicitly prove the dual-dilemma situation in the ‘double-layer’ game, we proposed the SED indicator, which quantifies whether the dynamics develop a social dilemma structure. This indicator is measured by the gap between the NE and the social optimal state. The dilemma strength [64–66], which explains the dilemma structure in simple two-player and two-strategy (2 × 2) games, is too simplistic for realistic dilemma games with substantially complex and time-dependent structures, such as vaccination games and traffic flow analysis. However, SED can quantify whether a game intrinsically has a social dilemma or not. We applied the SED to the present social dual-dilemma game, in which both vaccination and treatment dilemmas are inevitable.

## Supplementary Material

Code

## Data accessibility

This article has no additional data. The supplementary material provides the basic code of our study.
