# Supplementary material for: Modelling and analysing the coexistence of dual dilemmas in the proactive vaccination game and retroactive treatment game in epidemic viral dynamics
Source: Proc Math Phys Eng Sci. 2019 Dec 4;475(2232):20190484. doi: 10.1098/rspa.2019.0484 (PMC6936617; doi:10.1098/rspa.2019.0484)
Supplement: Code [file rspa20190484supp1.pdf]

```cpp
// SITR/V epidemic vaccination model.cpp' Prepared by K M ARIFUL Kabir. Email-
k.ariful@yahoo.com
//
#include "stdafx.h"
#include <iostream>
#include <fstream>
#include <stdio.h>
#include <math.h>
#include <vector>
#include <list>
#include <string.h>
#include <stdlib.h>
#include <ctype.h>
#include <sstream>
#include <string>
#include <cstdio>
#include <time.h>

using namespace std;

int main()
{
# define times 500
# define GENERATION 1000
# define k 0.1
# define Ro 2.5 //reproduction number


	ostringstream file1;

	file1<<" IB-RA model FES"<<".csv";

	ofstream Data1(file1.str().c_str(),ios_base::out|ios_base::trunc);

	Data1<<"es, e, FES"<<endl;


	ostringstream file2;

	file2 << " IB-RA model VC" << ".csv";

	ofstream Data2(file2.str().c_str(), ios_base::out | ios_base::trunc);

	Data2 << "es, e, VC" << endl;


	ostringstream file3;

	file3 << " IB-RA model FTR" << ".csv";

	ofstream Data3(file3.str().c_str(), ios_base::out | ios_base::trunc);

	Data3 << "es, e, FTR" << endl;


	ostringstream file4;

	file4 << " IB-RA model ASP" << ".csv";
```

```
ofstream Data4(file4.str().c_str(), ios_base::out | ios_base::trunc);

Data4 << "es, e, ASP, Rs, Rr, P" << endl;


double X[GENERATION + 1]; //fraction of vaccinators

double pi[GENERATION + 1]; // average social payoff

double S[times+1]; // susceptible

double V[times + 1]; //vaccinated

double V1[times + 1];

double NV[times + 1]; //non-vaccinated

double IUS[times+1];

double IUR[times + 1];

double T[times + 1]; //treatment

double R[times+1]; //recovered

double RSI[times + 1];

double RRI[times + 1];

double P[times + 1];

double T1[times + 1];

double Rc,B,G,Bs,Br,Gs,Gr,ms,mr,d,w,ls,lr,Rs,Rr,bb,Cr,e;

double piC, piD, Cts, Ctr,v,ls0,lr0,es,t1, xx1,pp1,ls1,lr1,ls2,lr2,ASP,Bt;

double eF1, eF2, eF3, eF4, eP1, eP2, eP3, eP4, ePP1, ePP2, ePP3, ePP4, ePP5, ePP6,
ePP7, ePP8, ePPP1, ePPP2, epiC, epiD;

Bs = 0.25;//infection rate of sensitive
Br = 0.2; //infection rate of resistant
Gs = 0.1; //recovered rate of sensitive
Gr = 0.1; //recovered rate of resistant
ms = 0.000001; //mutation rate (sensitive)
mr = 0.000001; // mutation rate (resistant)
d = 0.3;// delta; natural recovery rate
w = 0.3;  //prescribing probability
es = 0.5;  // treatment efficacy
Cts = 0.1; // relative cost of treatment (S)
Ctr = Cts; // relative cost of treatment (r)
Cr = 0.1; // relative cost of vaccine
Bt = 0.3; //cost of benefit


for (int cc = 0; cc< 100 + 1; cc++) {//efficiency loop
```

```
            es = cc / 100.0;

            for (int t = 0; t < 100 + 1; t++) {//effectiveness loop

                   e = t / 100.0;

            for (int j = 0; j < 100 + 1; j++) {//generation loop

                          X[0] = 0.5;

                          S[0] = 1 - X[j] - 0.00002;
                          V[0] = X[j];
                          IUS[0] = 0.00001;
                          IUR[0] = 0.00001;
                          T[0] = 0.0;
                          R[0] = 0.0;
                          RSI[0] = 0.0;
                          RRI[0] = 0.0;
                          V1[0] = 0.0;
                          NV[0] = 0.0;

                          T1[0] = 0.0;

                          P[0] = 0.001;

                   for (int i = 0; i < times; i++) {//time loop

S[i + 1] = S[i] - Bs * S[i] * (IUS[i] + (1 - es)*T[i]) - Br * S[i] * (IUR[i] + es *
T[i]);

V[i + 1] = V[i] - Bs * (V[i] - e * V[0]) * (IUS[i] + (1 - es)*T[i]) - Br * (V[i] - e *
V[0]) * (IUR[i] + es * T[i]);

IUS[i + 1] = IUS[i] + Bs * S[i] * (IUS[i] + (1 - es)*T[i]) + Bs * (V[i] - e * V[0]) *
(IUS[i] + (1 - es)*T[i]) - w * P[i] * IUS[i] - Bs * ms*S[i] * (IUS[i] + (1 - es)*T[i]) +
Br * mr*S[i] * (IUR[i] + es * T[i]) - Gs * IUS[i];

IUR[i + 1] = IUR[i] + Br * S[i] * (IUR[i] + es * T[i]) + Br * (V[i] - e * V[0]) * (IUR[i]
+ es * T[i]) - w * P[i] * IUR[i] + Bs * ms*S[i] * (IUS[i] + (1 - es)*T[i]) - Br * mr*S[i]
* (IUR[i] + es * T[i]) - Gr * IUR[i];

T[i + 1] = T[i] + w * P[i] * IUS[i] + w * P[i] * IUR[i] - d * T[i];

R[i + 1] = R[i] + Gs * IUS[i] + Gr * IUR[i] + d * T[i];

RSI[i + 1] = RSI[i] + Gs * IUS[i];

RRI[i + 1] = RRI[i] + Gr * IUR[i];

V1[i + 1] = V1[i] + Bs * (V[i] - e * V[0]) * (IUS[i] + (1 - es)*T[i]) + Br * (V[i] - e *
V[0]) * (IUR[i] + es * T[i]);

NV[i + 1] = NV[i] + Bs * S[i] * (IUS[i] + (1 - es)*T[i]) + Br * S[i] * (IUR[i] + es *
T[i]);

T1[i + 1] = T1[i] + w * P[i] * IUS[i] + w * P[i] * IUR[i];

ls1 = ((Bs*S[i] * (IUS[i + 1] + (1 - es)*T[i + 1])) + (Bs*(V[i] - e * V[0]) * (IUS[i + 1]
```

```
+ (1 - es)*T[i + 1])));

lr1 = ((Br*S[i] * (IUR[i + 1] + es * T[i + 1])) + (Br*(V[i] - e * V[0]) * (IUR[i + 1] +
es * T[i + 1])));

ls = ls1 / (ls1 + lr1);

lr = lr1 / (ls1 + lr1);

piC = ls * (-Cts+Bt-0.9) + lr * (-Ctr-0.9);

piD = ls * (-1+Bt) + lr * (-1);

ePPP1 = 1 / (1 + exp(-(piD - piC) / k));

ePPP2 = 1 / (1 + exp(-(piC - piD) / k));


P[i + 1] = P[i] - P[i] * ePPP1+(1 - P[i])*ePPP2;

Rs = (((Bs*d) + Bs * (1 - es)*w*P[i]) / (d*(w*P[i] + Gs)))*(S[0] + (1 - e)*V[0]);

Rr = ((Br*d) + Br * (es)*w*P[i]) / (d*(w*P[i] + Gr))*(S[0] + (1 - e)*V[0]);

                            }
eF1 = V[times];
eF2 = V1[times];
eF3 = S[times];
eF4 = NV[times];

pi[j] = -Cr * eF1 - (Cr + 1)*eF2 - eF4;
epiC = (-Cr * eF1 - (Cr + 1)*eF2)/(eF1+eF2); //Cooperators
epiD = -eF4/(eF3+eF4); //defectors
ASP = pi[j];

                            eP1 = 1 / (1 + exp(-(epiD + Cr) / k)); //Fermi PW
                            eP2 = 1 / (1 + exp(-(epiD + (Cr + 1)) / k));
                            eP3 = 1 / (1 + exp(-epiC / k));
                            eP4 = 1 / (1 + exp(-(epiC + 1.0) / k));

                            ePP1 = 1 / (1 + exp(-Cr / k));
                            ePP2 = 1 / (1 + exp(-(Cr - 1) / k));
                            ePP3 = 1 / (1 + exp(-(Cr + 1) / k));
                            ePP4 = 1 / (1 + exp(-Cr / k));
                            ePP5 = 1 / (1 + exp(Cr / k));
                            ePP6 = 1 / (1 + exp((Cr + 1) / k));
                            ePP7 = 1 / (1 + exp((Cr - 1) / k));
                            ePP8 = 1 / (1 + exp(Cr / k));

                            ePPP1 = 1 / (1 + exp(-(epiD - epiC) / k));
                            ePPP2 = 1 / (1 + exp(-(epiC - epiD) / k));

                            ///IB-RA///
X[j + 1] = X[j] - eF1 * ePP1*eF3 - eF1 * ePP2*eF4 - eF2 * ePP3*eF3 - eF2 * ePP4*eF4 + eF3
* ePP5*eF1 + eF3 * ePP6*eF2 + eF4 * ePP7*eF1 + eF4 * ePP8*eF2;//X update
```

```
           //SB-RA//

//X[j + 1] = X[j] - eF1 * eP1*(eF3+eF4) - eF2 * eP2*(eF3 + eF4) + eF3 * eP3*(eF1 + eF2) +
eF4 * eP4*(eF1 + eF2);//X update

                     }

Data1 << es << "," << e << "," <<R[times] << endl;

Data2 << es << "," << e << "," << eF1+eF2 << endl;

Data3 << es << "," << e << "," << T1[times] << endl;

Data4 << es << "," << e << "," << ASP << "," << Rs << "," << Rr << "," << endl;

              }
       }

       Data1.close();
}
```
